# Nasal mucosa micronuclei as early biomarkers of effect in workers exposed to airborne pollutants: a literature overview

**DOI:** 10.1007/s00204-025-04161-6

**Published:** 2025-09-01

**Authors:** Linda Ferrea, Paolo Durando, Guglielmo Dini, Alfredo Montecucco, Alborz Rahmani, Francesco D’Agostini, Sebastiano La Maestra

**Affiliations:** 1 Department of Health Sciences (DISSAL), University of Genoa, Via A. Pastore 1, 16132 Genoa, Italy; 2 Occupational Medicine Unit, IRCCS Ospedale Policlinico San Martino, Genoa, Italy

**Keywords:** Micronucleus assay, Nasal epithelium, Occupational exposure, Airborne pollutants, Biomonitoring, Genotoxicity

## Abstract

Occupational exposure to air pollutants poses a serious health concern for workers, particularly due to potential genotoxic effects. The micronucleus (MN) test is widely recognized as a reliable biomarker of early genetic damage. Although commonly applied to lymphocytes or buccal epithelial cells, the nasal mucosa, being the first site of contact for inhaled toxicants, has received relatively limited attention. This review aims to evaluate the scientific literature on the use of MN in exfoliated nasal epithelial cells as a biomarker of early genotoxic effects in workers exposed to air pollutants. We conducted a comprehensive literature search using PubMed and gray literature sources. Seventeen studies were identified that examined the frequency of MN in nasal cells of exposed workers, with or without comparison with other biomarkers, such as buccal or blood cells. Most studies have reported a significant increase in MN frequency in nasal cells following exposure to substances, such as FA, heavy metals, wood dust, and industrial chemicals. In some cases, nasal MNs appeared more sensitive than other cellular targets. However, data heterogeneity, lack of standardized protocols, and limited control for confounding factors (e.g., smoking, diet) hinder in-depth comparisons and meta-analyses. Nasal MN testing offers a promising, non-invasive tool for biomonitoring genotoxic exposure in occupational settings. However, further research is needed to develop standardized protocols, control for confounding factors, and clarify the relationship between MN frequency, exposure characteristics, and other biomarkers of effect.

## Introduction

### Biomarkers

Biomarkers are cellular, biochemical, or molecular alterations measurable accurately in a reproducible manner (Strimbu et al. 2010) in biological samples, such as cells, tissues, or fluids (Hulka et al. 1988), used to document the interaction of a hazard with biological systems (NRC 1987; WHO 1993). Moreover, biomarkers can be used to assess environmental, occupational, or dietary exposure to chemicals and determine dose–effect relationships for risk assessment, clinical diagnosis, and monitoring purposes.

In clinical settings, a diagnostic biomarker is used to classify, confirm, or evaluate the evolution of a disease (Califf 2018). In addition to the diagnostic field, biomarkers are increasingly being used in biomonitoring studies, as well as toxicological and epidemiological studies, to better define the causal relationships between exposure and risks of adverse effects (DeCaprio 1997). As defined by NRC (2006),"*the ultimate objective of biomonitoring is to link information on exposures, susceptibility, and effects to understand the public health implications of exposure to environmental chemicals*".

Monitoring is crucial in occupational medicine and hygiene research for developing risk assessments. It involves a program of repeated measurements taken over time and across different locations to quantitatively identify hazardous chemical substances (abiotic monitoring) and their metabolites in biological systems or in individuals who may be exposed (biomonitoring).

Environmental monitoring alone is insufficient in the occupational field to estimate the population's effect attributable to exposure. Specifically, although environmental monitoring can provide precise measurements of the xenobiotic being investigated, it underestimates individual responses because it does not consider physiologic and genetic variability. Integrating the biomonitoring study allows for taking into account individual responses, contributing to assessing the real risk of the essential worker, for example, in medical-health surveillance (Louro et al. 2019).

Implementing biomarkers offers a more comprehensive perspective than traditional toxicology studies, identifying intermediate events between exposure and disease development. This enables a better understanding of health risks, highlighting biomarkers'crucial role in proactive disease prevention.

Biomonitoring biomarkers are mainly divided into biomarkers of exposure, biomarkers of effect, and biomarkers of susceptibility. (WHO 1993).

Biomarkers of exposure could be a xenobiotic or its metabolite measured in a compartment within the organism. This type of biomarker is used to assess and confirm individual or population exposure to a xenobiotic and may provide a link between exposure and internal dosimetry.

Susceptibility biomarkers are indicators of an organism's response to exposure to a xenobiotic. Susceptibility biomarkers are crucial in understanding individual variability in response to chemical exposure. These biomarkers, especially genetic factors, significantly impact how the body processes and responds to various chemicals.

Finally, biomarkers of effect could instead be measurable behavioral, physiological, or biochemical alterations in the organism that can be associated with damage closely associated with the disease event. Together with exposure biomarkers, these biomarkers can define dose–response relationships highlighting adverse health effects or preclinical alterations determined by exposure and absorption of the xenobiotic (WHO 1993).

Effect biomarkers can be further subdivided into early or late. Specifically, early effect biomarkers are biological markers of events in the early phase of the interaction between the biological system and the xenobiotic. Early effect biomarkers include DNA adducts, epigenetic markers, enzyme inductions/inhibitions, sister chromatid exchange, DNA strand breaks, chromosomal aberrations, and an increase in the number of micronuclei (MN) (Tsitsimpikou et al. 2013; Weinhouse et al. 2015).

Late-effect biomarkers, on the other hand, measure the actual clinical disease, or in any case, the structural or functional alterations in the affected biological systems, so they fall into this class of chemical biomarkers, such as hormone levels, or a whole series of quantitative health markers, such as anogenital distance, behavioral tests, or even magnetic resonance imaging (Zare Jeddi et al. 2021;

Ventura et al. 2021; Rodriguez-Carrillo 2022).

Ideal biomarkers should be specific, sensitive, and proportional to the severity of the damage, early visible, easily accessible, analytically stable over time, the same between different species, associated with a known mechanism and be able to localize the damage as specifically as possible (Institute of Medicine US 2008).

Although discoveries of new biomarkers have been made in recent decades, numerous limitations are present to their use. Few biomarkers overlap with established standards, highlighting the potential for innovation in this field. For example, a key challenge is the need for non-invasive methods for collecting samples for analysis (Gupta et al. 2014).

### Workplace exposure

Workers in different occupational sectors are potentially exposed to hazards, including safety, biological, physical, ergonomic, chemical, and workload.

The exposure routes by which workers can contact hazardous substances include inhalation, skin contact, eye contact, ingestion and parenteral transmission, especially in workplaces like hospitals. When a subject is exposed to a risk factor, this can cause systemic, organ or local effects. The manifestation of the impact depends fundamentally on the chemical–physical nature of the agent with which the subject has come into contact.

For example, some chemical substances, in addition to having direct effects at the point of contact, such as irritation to the eyes, skin, mouth, or nose, can have adverse effects on other systems once absorbed, such as liver, blood, and nervous system. Moreover, many compounds can also generate secondary metabolites, the result of metabolic processes, which can modify the magnitude of the toxicity of the initial substance (Delclos and Lerner 2008; Croom 2012).

The main route of exposure to toxic substances is inhalation. About 70% of all deaths from occupational diseases are due both to inhalation cancer and respiratory diseases. These last can be linked to acute and chronic inhalation exposure to various airborne pollutants, such as chemicals, organic and inorganic particles, fibers, drugs, radioactive contaminants, dust, gasses, fumes, metals, and microbiological agents (WHO 2021).

Noteworthy, workers and their family members may be exposed to toxic chemicals at higher concentrations for extended periods than the general population, increasing their risk of acute and long-term health effects (U.S. EPA 2011). As reported by Locatelli et al., exposure to fumes, dust, gasses, and vapors during work is among the most important factors in the onset of respiratory diseases, such as asthma and chronic obstructive pulmonary disease (COPD) (Locatelli et al. 2023). Furthermore, industrial substances, such as wood dust, asbestos, arsenic, benzene, and vinyl chloride, are associated with the risk of developing cancer (NIOSH 1986), while occupational radon exposure appears to be associated with lung cancer death rates (Lubin 1995).

Many of these substances are known to be genotoxic, so they can potentially cause genetic alterations in the target tissues of exposed workers. (Nagalakshmi and Tong-man 1999).

Carcinogenesis is a multiphase process in which the exposure dose of a carcinogenic substance increases until it reaches the molecular dose sufficient to induce alterations such as DNA damage. DNA damage is preceded by various detoxification/metabolic activation mechanisms subject to individual variability and modulability by external factors, which makes the exposure dose different from the molecular dose. However, once the DNA damage has occurred, the cell activates a whole series of repair mechanisms and can choose to head toward cell death by apoptosis. In the case of not repaired DNA damage, the first stage of carcinogenesis, called initiation, was concluded, but in any case, to reach the complete formation of the tumor and the acquisition of malignancy, further mechanisms, such as promotion, progression, and metastasis, are necessary (De Flora et al. 2011).

Current estimates reveal that 3–6% of tumors globally are tied to occupational exposure, highlighting the urgent need to monitor workers’ exposure to hazardous substances (Hopf et al. 2019).

### MN as a biomarker of genotoxicity

Chronic degenerative diseases are characterized by a long latency period that occurs between exposure to various risk factors and health factors and the onset of the disease. Various molecular alterations may be present and detectable during this period to understand the evolution of the pathological process or the risk of getting sick (De Flora et al. 1996).

Environmental exposure to different substances, such as those listed above, can cause genotoxic damage, which is probably the leading cause of the development of chronic degenerative diseases, and minimally invasive and reliable biomarkers could improve biomonitoring techniques (Holland et al. 2008).

Biomarkers of genotoxic damage could be DNA adducts, DNA aberrant methylation, 8-hydroxydeoxyguanosine (8-OHdG), loss of FHIT function, nuclear and cytogenetic alterations (binucleated and multinucleated cells, MN, chromosomal aberrations, sister chromatid exchanges), DNA strand breaks, and apoptosis (D'Agostini and La Maestra 2024).

MN arise from excluding entire chromosomes, or fragments, from the nucleus following mitosis. The micronucleus test is one of the most widely used genotoxicity tests (Hayashi 2016; Sommer et al. 2020) for molecular epidemiological studies and is considered a biomarker of early biological effects (Fenech et al. 1999; Çelik et al. 2006; Bonassi et al. 2007; Knudsen et al. 2007; Holland et al. 2008; Samanta et al. 2012).

Moreover, different biomarkers, such as the comet test, can be considered valuable tools in genotoxic damage evaluation, with the difference that MN highlights irreparable chromosomal damage, while the comet test specifically measures DNA fragmentation, which may still be repairable (Maluf 2004).

MN formation results from losing acentric chromatids or chromosomes during cell division. These fragments can be lost in anaphase and outside the nucleus. The formation of MN can stem from a diverse range of mechanisms, including misattachment of tubulin, defects in kinetochore protein assembly, late replication, histone epigenetic changes, nucleoplasmic bridges generation, and gene amplification (Fenech et al. 2011). They can arise spontaneously as a vital process for extricating excess DNA (Suzuki et al. 2008) or during the normal development of progenitor cells (Peterson et al. 2011). Additionally, MN formation can be correlated to different stressogenic factors, such as oxidative stress or environmental exposure to clastogens or aneugenic substances (NRC 2006; Iarmarcovai et al. 2007; Kirsch-Volders et al. 2011). Many genotoxic agents alter DNA replication, interfering with the mitotic spindle or different cell cycle checkpoints. This interference can lead to incorrect mitosis, also contributing to the formation of binucleated or multinucleated cells (Bahar et al. 2002; Lens and Medema 2019).

MN test has been widely used to determine genotoxic damage in the biomonitoring of classes of populations exposed to mutagens and carcinogens, both physical and chemical. Many studies show a statistically significant increase in the frequency of MN in exposed subjects compared to controls (Holland et al. 2008).

In particular, the sensitivity and specificity of the micronucleus test vary depending on the conditions in which the study is performed. Different studies attributed a sensitivity of the micronucleus test between 70 and 90%. The quality of the sample and the accuracy in reading the cells seem to be the principal factors influencing the test sensitivity. On the other hand, the MN test specificity is generally higher (85–95%), but this also varies based on the quality of the sample and the experience of the operator who reads the results.

The reliability of the test can vary based on the protocol used, the population exposure, and the analytical methodology applied. In this context, the value of MN for performing radiation biodosimetry has been known for many years (Thierens and Vral 2009). In any case, the micronucleus test is considered one of the most reliable tools for detecting chromosomal damage and genetic abnormalities, even if it depends a lot on the correct interpretation of the results, requiring a certain amount of experience on the part of the operators.

## Cellular target

MN can be investigated in various organs, tissues, and body fluids, and their frequency depends on biological, environmental and lifestyle factors, which are the main determinants of individual susceptibility (Luzhna et al. 2013). Moreover, although MN tests can be performed in all nucleated cells, sampling specific organs can require invasive procedures, making biomonitoring studies inadvisable. This obstacle can generally be overcome by testing surrogate cells in which an inaccessible target cell can be explored by analysis of accessible or surrogate cells, such as peripheral blood lymphocytes.

Concerning biomonitoring studies referring to inhalable pollutants exposure, different cell targets, such as blood cells, alveolar macrophages, and epithelial cells, can be used to assess the early effect, referred to DNA damage.

In particular, analysis of lymphocyte DNA damage can be detected by the cytokinesis-block micronucleus cytome assay performed on cultured lymphocytes obtained from subjects exposed to different risk factors. The method described by Fenech (Fenech and Morley 1985) involves the use of cytochalasin B to block cytokinesis, which allows only cells that have divided in culture to be assessed, increasing the sensitivity of the method (Hamurcu et al. 2001). The advantage of using lymphocytes for micronucleus testing is their extended stay in circulation, where they can be exposed to different genotoxic agents, even though the more significant disadvantage is that it requires an ex vivo nuclear division (Nersesyan et al. 2016). Although erythrocytes can also be used to determine MN, especially reticulocytes, in humans, this is impractical since splenic sorting effectively removes micronucleated erythrocytes from peripheral blood, significantly reducing the frequency of MN.

Considering exposure by inhalation and subsequent damage triggers in the respiratory system, alveolar macrophages (PAM) are excellent surrogate cells since they are in close contact with the target tissues. PAMs are abundant phagocytic mononuclear cells (3–15 million/g of the lung) resident in the alveolar area, playing a fundamental role against infections, antigens, pollutants and inhaled particles. In addition to their phagocytic action, they play a role in modulating the inflammatory response at the pulmonary level (Garbi and Lambrecht 2017; Woo et al. 2021) and in the physiopathology of many pulmonary diseases (Almatroodi et al. 2014; Puttur et al. 2019; Hetzel et al. 2021).

Large numbers of PAMs are easily recoverable by bronchoalveolar lavage (BAL), and different studies conducted on humans exposed to genotoxic agents in the workplace evaluate the biomarkers of genotoxicity on alveolar macrophages obtained by BAL (Davison et al. 1983; Demedts et al. 1984; Lewtas et al. 1993.). BAL is the only technique to obtain fluid from the airways and lung lining, providing information about lung injury. This technique involves bronchoscopy for segmental lavage, where a slow infusion of a saline solution is perfused into a specific bronchus to be gradually vacuumed (Hoffman 2008).

Bronchoscopy is an invasive technique that may not be safe and is not recommended for sensitive individuals (Kodavanti 2014). For this reason, a less-invasive technique, such as spontaneous or induced sputum collection, is preferred. This non-invasive procedure allows for the recovery of substantial quantities of PAMs. Recent advancements in this technique have significantly enhanced its efficacy, enabling researchers to obtain sufficient sputum samples from those who cannot spontaneously produce them (D’Agostini and La Maestra 2024).

Considering that 90% of tumors appear to have an epithelial origin (Cairns 1975), addressing the test on the epithelial exfoliate cells would be preferable. In case where assessment of DNA damage in the epithelium is not feasible, as may be the case in the lung, one strategy may be to use cells sampled from the upper airway. For example, the buccal and nasal mucosa are excellent cellular targets for determining the genotoxic effects triggered by inhalation of toxic substances. Moreover, sampling these cells requires less-invasive techniques than obtaining blood samples for lymphocyte assays (Holland et al. 2008) and does not require ex vivo nuclear division.

Other cells that could be considered in biomonitoring studies of the exposed population are urothelial. These can be easily collected from urine by simple centrifugation. These cells have a morphology similar to buccal epithelial cells and have been used to determine the damage due to exposure to various toxic agents, such as Cr, formaldehyde (FA), and cigarette smoke.

## MN in buccal and nasal cells

Concerning occupational exposure by inhalation, non-invasive buccal and nasal cells represent a good target for assessing harmful effects triggered by different factors, such as occupational and environmental exposure, lifestyle, radiotherapy, and chemotherapy.

First proposed in 1986 (Stich et al. 1983), the buccal cell MN test now finds numerous applications as a biomarker for genotoxic damage (Salama et al. 1999; Bonassi et al. 2005; Speit and Schmid 2006; Iarmarcovai et al. 2007; Ceretti et al. 2014; Feretti et al. 2014). This is described by standardized protocols by Thomas et al. (2009), which involve brushing out cheek cells with a toothbrush, staining them, and viewing them under the microscope. Despite being a quick, cost-effective, and straightforward method for assessing workers'exposure to genotoxic substances, unfortunately, confounding variables, such as smoking, alcohol, diet, consumption of hot drinks, and dental fillings that may influence the frequency of MNs in buccal cells have not yet been extensively addressed and quantified (Holland et al. 2008). Furthermore, the buccal cells do not always represent the first site of contact for the respirable substances that are inevitably conveyed through the nasal passages in humans.

DNA damage induced by volatile genotoxic compounds, to which the working class is exposed, is evaluated by assessing the frequency of MN in nasal cells, which constitute the normal gateway of the respiratory system (Burgaz et al. 2002; Sarto et al. 1990). Although nasal cells are the site of first contact for volatile compounds, few studies evaluate the genotoxic effects of inhalable substances on this cellular target.

Furthermore, given the absence of standardized protocols for this type of analysis (Bruschweiler et al. 2014), most of these studies were conducted in parallel with the analysis of the buccal mucosa, which is currently one of the main targets for this type of evaluation, highlighting how these two analyses are often considered complementary. On the other hand, a study evaluating workers'exposure to welding fumes highlights how the analysis of MN on nasal cells may be more sensitive than that conducted on buccal cells (Wultsch et al. 2014).

The nasal mucosa is the first contact site in the inhalation exposure. Furthermore, nasal epithelium contains a variety of metabolizing enzymes necessary for the activation of promutagens, making them a promising target for the evaluation of genotoxic effects caused by occupational exposure by inhalation (Ye et al. 2005; Burgaz et al. 2002; Harkema et al. 2006). Moreover, Knasmueller et al. (2011) reported that nasal exfoliated cells would be more sensitive to MN evaluation than oral and blood cells.

Nasal cytology also allows not only the evaluation of DNA damage biomarkers such as MNs, but also the observation of any cytogenetic effects (binucleated cells), evaluation of the proliferative potential (basal cells) and cell death (condensed chromatin, pyknotic cells and karyolytic cells) (Bruschweiler et al. 2014). In this context, as reported in Table 1, the number of studies assessing occupational exposure using nasal cells is much lower than those conducted on other cellular targets. Although no standardized protocols for nasal MN tests have been published, the criteria for classifying buccal cell nucleus abnormalities can be used.

**Table 1:** Biomonitoring studies on different types and levels of exposure, duration of exposure, sample size with gender distribution, MN frequencies (mean ± SD) in nasal, buccal, and lymphocyte cells (when available), and smoking status

References	Exposure	Exp.- time	Sample size and gender		MN frequency ‰ ± SD						Smokers	
					Nasal		Buccal		Lymphocyte			
			Exp	Unexp	Exp	Unexp	Exp	Unexp	Exp	Unexp	Exp	Unexp
Burgaz et al., 2011	FA (2–4 ppm air, full work cycle)	2–4 years	23 (12 M; 11 F)	25 (M)	1.01 ± 0.62	0.61 ± 0.27	–	–	–	–	9	19
Ying et al. 1997	FA (0.520 mg/m^3^)	8 weeks	25	25	3.85 ± 1.48	1.20 ± 0.68	0.86 ± 0.56	0.57 ± 0.32	no effect (data not showed)		–	–
Ballarin et al. 1992	FA (0.1–0.39 mg/m^3^) + wood dust (0.23–0.73 mg/m^3^)	6.8 years ± 5	15 (8 M; 7 F)	15(8 M; 7 F)	0.9 ± 0.47	0.25 ± 0.22	–	–	–	–	0	0
Titenko-Holland et al. 1996	FA (0.153 ppm/die)	90 days	31(24 M; 7 F)	31 (7 M; 24 F)	2.5 ± 1.3	2 ± 1.3	2 ± 2	0.6 ± 0.5	–	–	5	5
Ye et al. 2005	FA (0.985 ppm/die)	8.5 years	18 (11 M; 7 F)	23 (12 M; 11 F)	2.7 ± 1.50	1.25 ± 0.65	–	–	–	–	0	0
	FA (0.107 ppm/die)	12 weeks	16 (4 M; 12 F)		1.89 ± 0.99		–	–	–	–	0	0
Suruda et al. 1993	FA (0.33 ppm/8 h)	85 days	29 (22 M; 7 F)	29 (22 M; 7 F)	0.50 ± 0.67	0.41 ± 0.52	0.60 ± 1.27	0.046 ± 0.17	6.36 ± 2.03	4.95 ± 1.72	5	24
Burgaz et al. 2002	Cr (exp. 4.43 µg/g–unexp. 0.28 µg/g creat.); Cb(exp 24.79 µg/g– unexp. 0.12 µg/g creat.); Ni (exp. 7.65 µg/g–unexp 2.42 µg/g creat.)	13.05 years	27 (M)	15 (M)	3.50 ± 1.80	1.19 ± 0.53	–	-	4 ± 2.98	1.4 ± 1.30	20	11
Huvinen et al. 2002	Cr^6^ (0.5 µg/m^3^)	21.4 Years	29	39	7.03 ± 3.57	7.85 ± 3.66	–	–	–	–	–	–
	Cr^3^ (248 µg/m^3^)	24.4 Years	14		6.79 ± 2.86							
	Cromite (22 µg/m^3^)	27.6 Years	5		7.6 ± 4.28							
Wultsch et al. 2014	Cr (2.31 µg/l whole blood)	–	22 (M)	22 (M)	0.55 ± 0.13	0.34 ± 0.1	0.31 ± 0.1	0.27 ± 0.07	–	–	9	10
	Cu (8.23 µg/g creat.)											
	Mn (14.68 µg/l whole blood)											
	Ni (3,97 µg/l urine)											
	Mo (0.008 µg/dl serum)											
	1-OHP (0.61 µg/g creat.)											
Wultsch et al. 2017	Cr^6^ (0.44 µg/l plasma)	9.7 years	42 (40 M; 2 F)	43 (41 M; 2 F)	no differences		no differences		–	–	21	23
	Co (0.85 µg/l plasma)											
Sarto et al. 1990	CrO_3_ (urinary Cr: > 2 µg/g creat.)	8 years	16 (15 M; 1 F)	27 (11 M; 16 F)	0.50 ± 0.78	0.44 ± 0.59	0.23 ± 0.35	0.51 ± 0.43	–	–	8	10
	EtO < 0.38 ppm	5 years	9 (F)		0.77 ± 0.53		0.48 ± 0.47				1	
	EtO acute exposure		3 (F)		2.32 ± 1.74						-	
Wultsch et al. 2015	wood dust and organic glue	23.2 years	38 (35 M; 3 F)	65 (43 M; 22 F)	no differences		no differences		–	–	6	22
	wood dust and VOC	15.7 years	51 (25 M; 26 F)						–	–	27	
Bruschweiler et al. 2014	wood dust 2.9 mg/m^3^	20 years	31 (M)	19 (M)	3.2 ± 2.2	0.9 ± 0.8	2.8 ± 1.5	–	–	–	–	–
Godderis et al. 2004	styrene: 9.5 ppm in air	12.5 years	44 (M)	44 (M)	0.52 ± 0.49	0.23 ± 0.31	–	–	3.93 ± 2.75	2.65 ± 1.94	–	–
	urinary mandelic acid: 201.75 mg/g creat											
Demircigil et al. 2010	SiO₂	40 h/weeks for 6.82 years	50 (M)	29 (M)	8.30 ± 2.35	2.84 ± 1.61	–	–	12.48 ± 4.17	5.59 ± 2.86	24	18
Wultsch et al. 2013	Ammonia: 0.76 mg/m^3^	3 weeks	25 (M)	21 (M)	no differences		no differences		–	–	6	6
	Nitrogen monoxide: 0.63 mg/m^3^											
	Nitrogen dioxide: 0.30 mg/m^3^											
	Hydrogen sulfide: < 0.45 mg/m^3^											
	Endotoxins: 382 EU/m^3^											
Wultsch et al. 2019	Street marking materials	6.6 h/day for 14 years	42 (M)	42 (M)	23% increase in MN frequency (exposed vs. controls)		34% increase in MN frequency (exposed vs. controls)		–	–	33	21

Exposure levels are expressed as environmental concentrations (e.g., ppm or mg/m^3^) or biological indicators (e.g., µg/g creatinine, µg/L whole blood)

"Exp."= exposed group;"Unexp."= unexposed control group;"–"= data not available;"No difference"= no statistically significant difference reported between groups

## Discussion and conclusions

The MN assay is widely used due to its simplicity, cost-effectiveness, rapid results, and high accuracy. Furthermore, this highly sensitive technique can detect whole chromosomes or chromosome fragments in the cytoplasm of eukaryotic cells, offering clear evidence of genetic material damage. Although historically, most genotoxicity studies focus on lymphocytes as a cellular target for the assessment of MN, based on the assumption that lymphocytes are valid indicators of systemic damage triggered by exposure to different pollutants, the use of epithelial cells could guarantee a more real assessment of exposure risk. In fact, epithelial cells exhibit different characteristics, including active metabolism, rapid turnover, and the ability to proliferate, exposing them to various cell cycle stages. In particular, when referring to airborne pollutants, the first contact occurred with nasal mucosa cells that are characterized by turnover that was calculated to be long, about 20–80 days (Basbaum and Jany 1990), differently to buccal cells that were established about 5–6 days (Harris and Robinson 1992; Shojaei 1998). A longer cell turnover time involves prolonged exposure to respirable pollutants. This prolonged contact may increase the probability of detecting cellular damage that has been fixed during the exposure period.

Furthermore, significant histological differences exist between the buccal epithelium, commonly used in biomonitoring studies, which typically involves cheek epithelial cells, and the nasal mucosa epithelium. The buccal epithelium primarily comprises semi-keratinized cells, which resist the mechanical abrasion caused by chewing and contact with food and teeth. In contrast, the nasal mucosa comprises a ciliated pseudostratified columnar epithelium, with goblet cells that secrete mucus and lack keratinization. These histological features make nasal cells more vulnerable to environmental stressors, including PM, as they do not possess the protective barrier provided by keratin. Together, these considerations could explain why spontaneous MN nasal cells could be more abundant than buccal epithelial cells (Moore et al. 1993). On the other hand, MN in buccal cells can be induced by various factors, including consuming alcohol, hot beverages, dental implants or fillings, smoking, and poor oral hygiene.

Analysis of the literature highlights how 11 of the 17 studies reported an increase in the presence of MN in the nasal cells of workers exposed to airborne pollutants with genotoxic action.

In particular, 6 studies evaluated the relationship between occupational exposure to FA and MN. FA is a volatile chemical compound that is carcinogenic, an irritant, and can damage the upper respiratory tract and eyes when inhaled (Park et al. 2022). Several categories of workers are exposed to FA, including hairdressers, morgue workers, and solvent producers (Lindström et al. 2021).

The effects determined by exposure to this compound have been supported by almost all studies (Burgaz S et al. 2001; Ying et al. 1997; Ballarin et al. 1992; Ye et al. 2005; Suruda et al. 1993) except the one conducted by Titenko-Holland et al. (1996), who did not report a significant increase in MN in nasal cells following exposure to FA. Although in some studies, nasal mucosa, buccal mucosa, or lymphocytes are analyzed simultaneously, it is impossible to clarify which cell type is more sensitive to exposure. This can be ascribed to the different exposure times and concentrations using a non-standardized protocol.

Another 5 studies instead evaluate exposure to different heavy metals, including Cr, which in its hexavalent form is considered carcinogenic (Skowroń and Konieczko 2015). Only 2 of these studies were able to detect an increase in the frequency of MNs in nasal cells (Burgaz et al. 2002; Wultsch et al. 2013), while the others did not find any difference compared to the control group. Furthermore, the data reported by Wultsch et al. (2013) demonstrate how only the nasal mucosa cells of exposed personnel had an increase in MNs compared to buccal cells.

However, Sarto et al. (1990) observed an increase in the number of MNs only in the nasal mucosa in a group of workers accidentally exposed to high levels of EtOH, unlike buccal cells.

Further, two studies evaluated exposure to wood dust as capable of triggering adverse effects at the pulmonary level as a function of the frequency and duration of exposure (Schlunssen et al. 2018). The study conducted by Wultsch et al. (2015) did not find statistically significant differences in MN formation in nasal and buccal cells. On the contrary, Bruschweiler et al. (2014) reported a substantial increase in MN frequency in nasal and buccal cells compared to the control group, which showed a more significant increase in the buccal cells.

A study conducted by Godderis et al. (2004) highlights an increase in MN frequency in nasal cells in a group of workers exposed to styrene, a possible carcinogen included in group 2B (Pleban et al. 2017), compared to the control highlighting how the fold variation is more significant in the nasal mucosa cells.

Evaluation of genotoxic damage in workers in industries using sand, soil, and rocks and therefore exposed to silica dust (Demircigil et al. 2010; Wultsch et al. 2019) shows a statistically significant increase in nasal MN compared to control groups.

Currently, no standardized protocol for determining MN in nasal mucosa has been established as an early biomarker of effect in workers exposed to air pollutants.

The lack of uniform guidelines for data collection, analysis, and interpretation makes comparing the results obtained from different studies significantly difficult. A unified methodological approach would be crucial to ensure the reproducibility and validity of the results in different contexts, facilitating the application of biomarkers in the occupational setting and their integration into worker health monitoring programs.

Despite the growing interest in using MN as biomarkers of pollutant exposure, the scientific literature on the topic remains limited. The available studies are characterized by considerable heterogeneity in exposure conditions, protocols adopted and groups of workers involved. These differences compromise the possibility of drawing general conclusions on the value of these biomarkers since the observed effects could be influenced by variables related to the type of pollutant, the duration and intensity of exposure, and the demographic characteristics of the subjects. Therefore, it is necessary to standardize the protocols and conduct studies that control these variables more accurately.

Another critical aspect concerns the lack of studies comparing MN with other potential biomarkers of cellular damage or exposure to air pollutants. Comparing MNs with other biological indicators, such as DNA damage or inflammatory response, could provide a more complete view of the potential biological effects of exposures. Furthermore, this approach would allow the evaluation of the specificity and sensitivity of MNs compared to other biomarkers, facilitating a better interpretation of the collected data and promoting the identification of more effective monitoring tools.

Currently, there is a lack of in-depth understanding of the potential use of MNs as biomarkers for the chemical–physical characteristics of xenobiotics. The chemical and physical nature of pollutants, such as their solubility, stability, and ability to interact with cells, may influence the biological response and the formation of MNs. Studies conducted so far have not adequately explored how these characteristics may modulate the sensitivity of the biomarker, nor how they may affect the observed cellular responses. Therefore, it would be necessary to develop research that considers these aspects to optimize the use of MNs as specific exposure biomarkers.

Another relevant limitation in research on exposure biomarkers is the limited attention given to confounding factors that may influence the results. Variables, such as age, sex, pre-existing health status, and other possible exposures to toxic agents unrelated to the pollutant studied, are often overlooked in existing studies. The lack of adequate control for these factors may compromise the reliability of conclusions and limit the applicability of the results. Including these confounding factors in statistical analyses can improve the data's validity and precision and ensure that the identified biomarkers are associated with exposure to pollutants, not external uncontrolled variables.

Nasal micronucleus testing is a promising, minimally invasive method for the early detection of genotoxic damage in workers exposed to air pollutants. Compared to other cellular targets, such as buccal epithelial cells or peripheral lymphocytes, nasal epithelial cells may offer greater specificity for inhaled substances due to their anatomical location, metabolic activity, and longer turnover time.

However, heterogeneity in study designs, protocols, exposure conditions, and control for confounding factors limits the comparability and generalizability of existing results. Currently, there are no validated or standardized protocols for collecting, evaluating, and interpreting nasal micronucleus data, which represents a significant obstacle to its widespread application.

In conclusion, although preliminary evidence supports the use of nasal micronuclei in nasal epithelial cells as an early marker of occupational genotoxicity, rigorous validation through standardized longitudinal studies would be necessary to promote regulatory acceptance.
